# Potential Contribution of Climate Change to the Protein Haze of White Wines from the French Southwest Region

**DOI:** 10.3390/foods10061355

**Published:** 2021-06-11

**Authors:** Grégory Pasquier, Carole Feilhes, Thierry Dufourcq, Olivier Geffroy

**Affiliations:** 1 Université de Toulouse, INP-PURPAN, 75 Voie du TOEC, BP57611, CEDEX 3, 31076 Toulouse, France; olivier.geffroy@purpan.fr; 2 Institut Français de la Vigne et du Vin–V’Innopôle Sud-Ouest, 1920 Route de Lisle-sur-Tarn, 81310 Peyrole, France; carole.feilhes@vignevin.com (C.F.); thierry.dufourcq@vignevin.com (T.D.)

**Keywords:** must, wine, prediction, autochthonous cultivars

## Abstract

The aim of this study was to evaluate the role played by climatic conditions during grape ripening in the protein instability of white wines produced in the French southwest region. For this purpose, basic wine analyses were carried out on 268 musts and the corresponding wines, all produced during the 2016, 2017, 2018, and 2019 vintages, with distinctive climatic conditions. Qualitative and quantitative variables were correlated with levels of protein haze determined by heat test (80 °C/2 h) in the wines using analysis of covariance (ANCOVA), principal component analysis (PCA), and classification and regression trees (CART). Our results show that the climatic change, with the increase in temperatures, and the decrease in precipitation during the grape ripening phase, tends to enhance the risk of protein instability in wines. Indeed, the values of pH, titratable acidity, and malic acid concentrations of the musts, which are good indicators of the conditions in which the grapes ripened and of the level of ripeness of the grapes, were also the variables that correlated best with the protein haze. By measuring these parameters at harvest before alcoholic fermentation, it may be possible to predict the risk of protein haze, and thus early and precisely adapt the stabilization treatment to be applied.

## 1. Introduction

All the wine regions worldwide are experiencing the impact of climate change. Since the 1990s, its consequences have been widely studied and numerous peer-reviewed scientific journals have published articles on the subject. Indeed, global warming is now a fact, with an average increase of 1 °C compared to the pre-industrial revolution [1]. Temperatures became warmer during vine growth and grape ripening, leading to an advance in phenology and harvest date [2]. In addition, climate change is leading in most cases to a decrease in rainfall and/or an increase in evapotranspiration, resulting in increasingly marked vine water deficits [3]. Finally, stratospheric ozone depletion increases the intensity of UV radiation at ground level, leading to a modification of the vine’s physiology [4].

Climate evolution is contributing to a change in grape composition [5], resulting in wines that have nothing in common with those produced during the last century. Indeed, ethanol concentrations in wines have increased by 1 to 2% *v*/*v* in the past 40 years due to higher grape sugar content [6]. Conversely, titratable acidity declines while pH rises as a result of increasingly high temperatures during the ripening of the grapes [7]. This is particularly due to the decrease in malic acid, and the increase in potassium, which is involved together with tartaric acid in potassium bitartrate precipitation [8]. Finally, excessive temperatures (>30 °C) slow down the biosynthesis of anthocyanins in red grapes, and the formation of free and bound aromas in white grapes [9].

One of the consequences that, to our knowledge, has never been investigated is the impact of climate change on the protein instability of white wines. However, there is a lot of evidence in the scientific literature that a close link exists. The cloudiness that can occur in wines stored under inappropriate temperature conditions is mainly due to chitinases and thaumatin-like proteins (TLP) [10]. Indeed, the concentration of these proteins in wines is a key element of the protein haze formation [11,12]. For chitinase, increased concentrations are known to promote protein haze in wine [12]. The role of TLPs is more complex as such proteins may interact with other wine compounds such as polyphenols and polysaccharides [13]. These two families of pathogenesis related (PR) proteins continuously accumulate from veraison and throughout the ripening phase of the grapes. The later the grapes are harvested, the richer they are in chitinase and TLP [14]. In addition, high temperatures and UV-B radiation in the bunch area as well as a limited vine water supply favor their accumulation in grapes [15,16,17].

Other factors inherent to the wine composition play a role in the mechanism of protein haze. One of the most important of these is pH. Indeed, the charge of proteins and their interactions with other compounds depends on the wine pH, with larger protein instability being observed in high-pH wines [18,19,20].

Occitania is France’s leading wine region in terms of surface area (270,000 ha in 2020) and production volume (15.5 million hectoliters in 2020), which represents one third of the volume of French wines (i.e., 5% of world production). Due to its Mediterranean or Atlantic climate characterized by summer water shortages, it is particularly affected by climate change.

Various predictions suggest that the effects of climate change will become even more pronounced in the near future [21,22], so it is necessary to anticipate their consequences. The aim of this work, conducted on a set of musts and wines made from varieties planted in the southwest wine-growing area of the Occitania region, and produced during vintages with different climatic conditions, is to understand the relationship between simple oenological indicators and protein instability.

## 2. Materials and methods

### 2.1. Climatic Conditions

To precisely characterize the four studied vintages (2016–2019) from a climatic point of view, the level of precipitation and daily air temperatures (minima, maxima and average values) were provided by Météo-France (Toulouse, France). The musts and wines used for this study were sourced from two different geographical areas (Tarn and Gers) in the southwest wine-growing area. Climatic data were collected between 1997 and 2019 from the Lisle sur Tarn and Courrensan weather stations for the Tarn and Gers areas, respectively. Both stations are located within 20 km of the sites where the grapes were harvested. The data were used to calculate the Huglin Index (HI)—$\overset{30/09}{\underset{01/04}{\sum }}[\frac{\left(T_{average\;temperatures\;\left({}^{\circ}C\right)}-10\right)+\left(T_{maximum\;temperatures\;\left({}^{\circ}C\right)}-10\right)}{2}]*d_{\left(\mathrm{coefficient}\;\mathrm{for}\;\mathrm{the}\;\mathrm{average}\;\mathrm{daylight}\;\mathrm{period}\;\mathrm{in}\;\mathrm{the}\;\mathrm{studied}\;\mathrm{latitude}\right)}\;—$[23], cumulative rainfall (CR) between 1 April and 30 September, and between 1 January and 31 December. For each vintage, phenological dates were estimated from average phenological dates for each studied cultivar provided by the French Chambers of Agriculture and the French Institute of Vine and Wine (IFV). This information enabled us to estimate the date of veraison and thus to calculate CR from flowering to veraison (1 April–31 July) and from veraison to harvest (1 August–30 September).

### 2.2. Musts and Wines

The 268 musts and the 268 corresponding wines were produced in the experimental winery of IFV (Caussens and Lisle sur Tarn, France). They were made from vines planted in the two main white wine producing regions of the southwest, the Gers and the Tarn areas. Details on the musts and wines analyzed in this study are provided in Table 1. The vinification operations were carried out according to standard procedures validated in the IFV’s experimental cellar. Most of the wines were made according to the following winemaking scheme.

Grapes (60 kg) were hand-picked at commercial harvest (°Brix/Titratable acidity ratio), de-stemmed, and crushed with modern vibrating equipment (Le Cube, Socma, Narbonne, France). The grapes were then pressed using a modified pneumatic press (Marchisio, Vertova, Italy) for 600 s at 2 bars. The musts obtained received a 50 mg/L sulfur dioxide addition using a 10% bisulfite liquid solution (Solution 10, Laffort, Bordeaux, France) and a sample was collected at this time to perform the analysis. Musts were then introduced into a 30 L stainless steel beer keg, cooled down to 0 °C, and kept at this temperature for 72 h to allow a clarification level between 150 and 200 nephelometric turbidity unit (NTU). The turbidity was monitored with a 2100 AN IS turbidimeter (Hach, Loveland, USA). After increasing the temperature to around 15 °C, the musts (25 L) were inoculated with 200 mg/L of rehydrated active dried *Saccharomyces cerevisiae* yeast (Zymaflore VL3, Laffort, Bordeaux, France) and 300 mg/L of diammonium phosphate, previously dissolved in a small amount of must, was added. The musts were fermented without replicates at 18 °C. After alcoholic fermentation (less than 2 g/L glucose/fructose), all wines were racked and 60 mg/L sulfur dioxide was added. The wines were stored in the cellar for three months at ambient temperatures, typically not exceeding 8 °C during the winter period. Before bottling, the wines were cold-stabilized to avoid crystallization of tartaric salts (one month at 0 °C) but did not receive any bentonite addition. The wines were filtered through a cartridge filter (Pall France, Saint Germain-en-Laye, France) equipped with 5 and 1 µm filtration cartridges (Prédel, Saint Loubès, France). Free sulfur dioxide was adjusted to 25 mg/L and carbon dioxide between 600 and 650 mg/L at bottling. The wines were then bottled in 750 mL bottles, closed with screw caps, and stored at 12 °C before analysis.

For some wines, the general protocol described above was slightly adapted. These changes were mostly confidential and could include the level of SO_2_ addition, the duration of skin contact, the yeast strain, or the temperature of fermentation.

### 2.3. Conventional Enological Analysis in Must and Wine

After centrifugation (14,000× *g* for 6 min) with a ROTINA 420 R centrifuge (Hettich, Switzerland), sugar concentration (°Brix) of the must was determined using a PAL digital pocket refractometer (Atago, Japan), and pH using a Titromatic pH meter (Hachlange, Düsseldorf, Germany). Titratable acidity (g/L expressed as tartaric acid equivalent) was measured according to the OIV method [24]. A Konelab Arena 20 sequential analyzer (Thermo Electron Corporation, Waltham, USA) associated with enzyme kits was used to determine the contents of alpha amino nitrogen (α-NH_2_), ammonium (NH_4_^+^) (Megazyme, Ireland), and malic acid (Thermo Fisher Scientific, Waltham, USA). The determination of potassium was carried out by flame photometry (Bio Arrow, France) following the OIV method [24]. Tartaric acid was determined by colorimetric titration [25] and polyphenol contents were measured by means of the total polyphenol index (TPI), which corresponds to the 280 nm absorbance value, after dilution (1:10 *v*/*v*), using an Evolution 100 spectrophotometer (Thermo Electron Corporation, Waltham, USA). Apart from sugar concentration, the same analyses were carried out for the wines one month after bottling. In addition, alcohol content was measured with an Alcolyzer Wine (Anton Paar, Graz, Austria) and volatile acidity (g/L expressed as acetic acid equivalent) using a Konelab Arena 20 sequential analyzer combined with enzyme kits (Megazyme, Wicklow, Ireland). Free and total sulfur dioxide levels were measured using the iodometric method proposed by the OIV (2009). All determinations were performed in duplicate.

### 2.4. Heat Test

The heat stability of the wine samples was assessed using a protocol adapted from Pocock and Waters [26] and McRae [27]. All measurements were performed in triplicate on wine samples from the same bottle. Briefly, after measuring the initial turbidity of each wine, expressed in NTU, with a 2100 AN IS turbidity meter (Hach, Loveland, CO, USA), 20 mL of wine was placed in a test tube closed with a stopper. Samples were then heated to 80 °C for 2 h in a DHG-9053A fan oven and cooled to 4 °C for 16 h. Turbidity was measured again after homogenization, and room temperature acclimatization for 1 h. The calculated difference in turbidity before and after heating (ΔNTU) of the wine sample was regarded as haze intensity.

### 2.5. Statistical Treatments

Statistical analyses were conducted with XLSTAT software (Addinsoft, Paris, France).

#### 2.5.1. Impact of Climate Conditions on Protein Instability and Basic Must and Wine Parameters

As grape variety is an important factor to explain differences in wine protein instability, only the varieties whose wines were simultaneously available in 2016, 2017, 2018, and 2019 were kept to investigate the impact of climatic conditions on haze formation. The wines were also selected on the basis of the origin of their grapes. They were harvested from the same plots and in most cases, the same growing strategy was used from one vintage to the next. After optimized Box–Cox transformation, 124 observations (*n* = 31 for each vintage) were retained. Data from Verdejo, Len de l’El, Sauvignon, INRA-8458, Gros Manseng, Alvarinho, Verdelho, Rkatsiteli, and Colombard were used for the treatment. In the first instance, an analysis of covariance (ANCOVA) was performed using protein instability as the quantitative dependent variable. The quantitative explanatory variables were the oenological parameters measured on the musts and wines as well as the climatic data. The qualitative explanatory variables were the grape varieties and vintages. The model incorporated the variety * vintage interaction. Fisher’s least significant difference test was used for post-hoc comparison of means at *p* < 0.05. In a second step, basic must (sugar (°Brix); titratable acidity (g/L tartaric acid); pH; tartaric acid (g/L); malic acid (g/L); alpha-amine nitrogen (α-NH_2_) (mg/L); mineral nitrogen (NH_4_^+^) (mg/L); potassium (K^+^) (g/L); Total Polyphenol Index (TPI)) and wine parameters (titratable acidity (g/L tartaric acid); pH; free SO_2_ (mg/L); total SO_2_ (mg/L); tartaric acid (g/L); malic acid (g/L); volatile acidity (g/L acetic acid); K^+^ (g/L); ethanol (% alcohol by volume (abv)); TPI) were used as quantitative dependent variables.

#### 2.5.2. Correlation between Basic Must and Wine Parameters and Protein Instability

To study the relationship between the 268 musts and corresponding 268 wines’ basic parameters and their protein instabilities (ΔNTU), a principal component analysis (PCA) was performed after data normalization (*n*) and using Pearson’s correlation at a significance level of α = 0.05.

#### 2.5.3. Relationship between Grape Variety Characteristics and Protein Instability

Classification and regression trees (CART) were carried out to create classes representative of the level of protein instability according to the basic must (*n* = 268) and wine (*n* = 268) parameters. This supervised machine learning algorithm showing a better adaptation to non-linear situations than partial least squares regression (PLSR) was preferred to other clustering methods. Cross-validation was performed by groups of 25 observations and the “Exhaustive CHAID” method at a 5% significance level was chosen. To limit overfitting, bias, and large variances, the conditions were set as follows: minimum size for a parent = 10, minimum size of a son = 9, and maximum depth = 5. To optimize the model, the qualitative variables ‘grape varieties’ and ‘vintages’, which reduced the bias to 1221 compared to 1425 for the initial model, were included.

## 3. Results and Discussion

### 3.1. Impact of Climate Conditions on Protein Instability and Basic Must and Wine Parameters

Among the four studied vintages (2016, 2017, 2018, and 2019), 2016 and 2018 were respectively the coldest and warmest. While the Huglin Index (HI) in 2016 (2248) was close to the average value calculated since 1997 for the Gers area (2235), it was below for the Tarn area (2085/2155). Aside from 2003, 2018 was the second hottest season since 1997 for both locations. 2017 and 2019 had similar HI in both areas, with values between the two vintages above-mentioned.

Cumulative rainfall (CR) between 1 April and 30 September also highlighted that 2016 and 2018 were the most extreme vintages among the four studied. However, this parameter differed between the Gers and the Tarn. Indeed, for Tarn, 2018 and 2016 were the least and most rainy vintage, with rainfall of 282 mm and 339 mm, respectively, while the opposite was observed for Gers (270 mm in 2016 and 402 mm in 2018). The CR between 1 April and 30 September for the Tarn area for the four vintages studied was close to or below the average value recorded since 1997 (337 mm). In the Gers area, 2018 was the only studied vintage that exhibited CR value above average data since 1997 (+32 mm).

**Figure 1:**
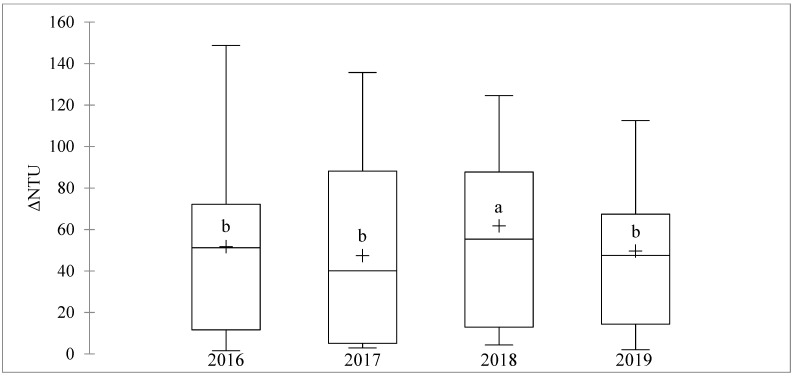
Box plots showing the median, range, and mean (+) of ΔNTU determined for white wines (*n* = 31) from Verdejo, Len de l’El, Sauvignon, INRA-8458, Gros Manseng, Alvarinho, Verdelho, Rkatsiteli, and Colombard grapes made during the four studied vintages (2016–2019). Different letters indicate means that are significantly different by Fisher test (*p* < 0.05).

Figure 1 illustrates the variability in ΔNTU measured on the 124 selected wines from the 2016, 2017, 2018, and 2019 vintages. The 2018 vintage had an average ΔNTU (61.8 NTU) significantly higher than the other three vintages. These were not significantly different from each other, although 2016 (51.8 NTU) tended to be higher than 2019 (49.6 NTU), which was higher than 2017 (47.5 NTU).

**Figure 2:**
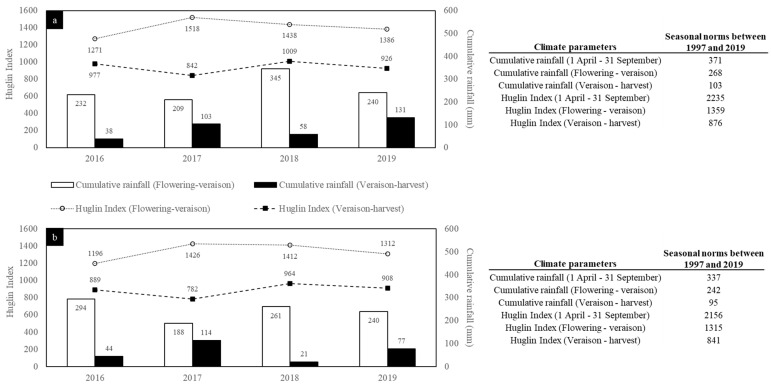
Climatic conditions observed in the Gers (**a**) and Tarn (**b**) areas.

As above-mentioned, the 2018 vintage was identified as the hottest of the four vintages. Focusing on the veraison–harvest period (Figure 2), it can be seen that the 2018 vintage was the warmest and one of the least rainy of the four studied. In contrast, 2017 was the coolest and one of the wettest over the same period. 2016 was characterized by cool weather during the flowering–veraison period, with a HI below the average calculated value since 1997 (HI = 1360 in the Gers area, and HI = 1315 in the Tarn area). For this latter vintage, HI and CR for the veraison–harvest period were close to those observed in 2018. 2019 is the vintage whose conditions of climate come closest to the average data calculated since 1997. The climatic data calculated for the Tarn and the Gers area differed in terms of absolute values, but their trends were quite similar and properly reflect the overall climate of the southwest of France during the four studied vintages.

It can be hypothesized that climatic conditions during the veraison–harvest period play an important role in the level of protein instability in white wines. Indeed, proteins responsible for the protein disorder (TLP and chitinase) mainly accumulate in grapes over this period [14] with a final concentration in grapes being highly dependent on climatic conditions during ripening. Grapes that ripen in hot conditions on vines that experience a limited water supply or are exposed to high UV radiation usually contain higher levels of TLP and chitinase [15,16,17,28,29,30].

The effect of climatic conditions on protein instability seems to be more marked than on basic parameters (Table 2). Indeed, the analytical markers, often put forward to assert that a wine was produced in a hot and dry climate, did not allow us to discriminate the four studied vintages. Notably, must sugar and alcohol contents were not significantly higher in 2018. Conversely, titratable acidity and malic acid levels, were not lower and pH higher in 2018 for which grapes ripened in warmer and dryer conditions. In this particular case, these parameters illustrate more technological decisions made during grape production (low leaf to fruit ratio for Colombard and Gros Manseng, which is not favorable to ripening, timing of harvest in relation with a desired sugar-acid balance, etc.) than a reflection of the climatic conditions during the ripening of grapes. However, it should be noted that for 2016 and 2018, two vintages with warm and dry conditions, the α-NH_2_ and NH_4_^+^ concentrations were lower. It has been well demonstrated that the synthesis of these nitrogenous elements, which are protein precursors, are generally affected by hot or dry weather conditions [31,32].

### 3.2. Correlation between Basic Must and Wine Parameters with Protein Instability

Table 2 presents the median, minima, and maximum values for the oenological parameters measured on all musts and wines. Must sugar concentrations varied between 14.9 and 24.6 °Brix and alcohol contents in wines between 9.6 and 15.4% abv. These values are consistent with those reported in the scientific literature [33,34,35]. Within the wine analysis pool, it should be noted that sugar levels close to 25 °Brix and alcohol contents above 15% abv were measured in musts and wines from the Gros Manseng grape variety produced in the Gers area. Although the median values fell within the classic range, the lowest values measured for titratable acidity in musts (3.33 g/L) and wines (3.75 g/L) are below those found in the literature [36,37]. In contrast, some musts and wines have very high pH values (>3.4), a threshold sometimes mentioned to describe white musts or wines made from over-ripe grapes [38]. These include the Voltis and Floréal varieties, which showed relatively high pH values (>3.5) in 2018 and 2019. The pH values generally found in wines are between 3 and 4 [38], with a range between 3 and 3.4 for white wines [37]. The median value of 3.1 measured in the musts and wines from this series is at the lower end of this range. These pH values depend particularly on the harvest date, which was imposed by commercial harvest. Furthermore, most of the samples came from the Gers area where yields are generally high (15 T/ha on average), involving low leaf to fruit ratios (<1) that are not favorable to obtain high levels of ripeness. Moreover, the musts and wines with the lowest pH (<2.7) were made also from Colombard and Gros Manseng from the Gers area. Finally, the concentrations of tartaric and malic acids found in the musts (1.71–6.38 g/L; 0.85–10.29 g/L) and wines (0.8–4.39 g/L; 0.43–8.10 g/L), respectively, are extremely low in comparison with the usual values [36,37,38,39].

Table 3 shows the Pearson’s correlation coefficients (*p* < 0.05) between the quantitative variables of the study. Sugar (*r* = 0.160), potassium (*r* = 0.145), pH (*r* = 0.544), and total polyphenol index values (*r* = 0. 219) measured in the musts were positively correlated with protein instability, whereas titratable acidity (*r* = −0.536), malic acid (*r* = −0.432) and NH_4_^+^ (*r* = −0.136) were negatively correlated. Wine pH (*r* = 0.476) and alcohol content (*r* = 0.133) were positively correlated while total acidity (*r* = −0.504), tartaric acid (*r* = −0.179), and malic acid (*r* = −0.389) concentrations showed a negative correlation. R², obtained by squaring the Pearson coefficient, provides the proportion of variability of a single variable that can be explained by the other. In our case, 28.7% of the protein instability of the white wines analyzed in this study was explained by the titratable acidity of their musts, 29.6% by their pH, 18.6% by their malic acid content, 25.4% by the titratable acidity of these wines, 22.7% by their pH, and 15.2% by their malic acid content.

This approach tends to show that the protein instability of white wines increases at higher pH and lower titratable acidity and malic acid concentration. Unstable wines are made from musts with the highest pH, and the lowest titratable acidity and malic acid concentration. The role of the acid–base balance of wines on their protein stability is well known [40,41,42]. It has been shown that when wines are placed at 25 °C, protein instability is greater at low pH than at high pH [19]. Conversely, when wines are placed at higher temperatures, protein instability is greater at higher pH than at lower pH [19]. Thus, considering the thermal tests applied in most oenology laboratories and wineries (80 °C) [10], the most unstable wines are those with the highest pH. As malic acid concentration and titratable acidity, which reflects the concentration of titratable protons, decrease, the protein instability tends to increase. These results are consistent with previously published results that highlighted that organic acids have a stabilizing role against protein flocculation [43]. Apart from the fact that organic acids can play a direct role in the protein instability, it should be mentioned that they reflect the conditions in which the grapes have grown and ripened. Indeed, pH, titratable acidity, and malic acid concentrations of musts are three of the most relevant indicators to monitor grape ripening [44].

The pH of musts and wines tends to increase with temperature while titratable acidity decreases [45]. The climatic conditions during the growth and ripening of the grapes are known to play an important role in the organic acid content of the must and wine [46]. This can be easily explained for malic acid as the activity of the grape malic enzyme, responsible for the catalysis of malic acid into pyruvic acid, increases as temperatures rise [47]. When temperatures and enzyme activity are higher during ripening, the grapes contain less malic acid.

Therefore, it is possible to hypothesize that the most unstable wines might come from (i) the ripest grapes or from grapes that ripened in the hottest conditions; (ii) grapes grown on a vine with a limited water supply; and (iii) grapes exposed to intense UV radiation.

Other variables such as sugar, potassium, alcohol, and polyphenol contents could support this observation. However, the correlations, whether positive or negative with protein instability, remain weak. Some of these variables such as alcohol, potassium, and polyphenol concentrations have already been mentioned as being able to influence haze formation [20,48,49]. In our experimental conditions, they did not seem to be as discriminating as pH, titratable acidity, and malic acid concentration.

### 3.3. Relationship between Grape Variety Characteristics and Protein Instability

**Figure 3:**
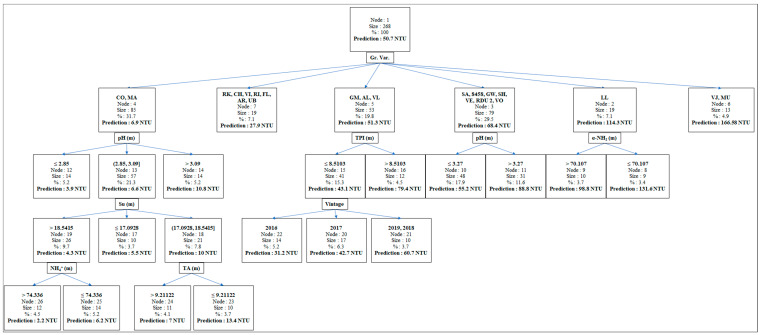
Classification and regression tree carried out with all the oenological parameters measured in the musts and wines and the “grape variety” and “vintage” quality variables. “Prediction” is the average protein instability (ΔNTU) calculated for each class. Gr. Var.: Grape Variety; CO: Colombard; MA: Mauzac; RK: Rkatsiteli; CH: Chardonnay; VI: Viognier; RI: Riesling; FL: Floréal; AR: Arvine; UB: Ugni blanc; GM: Gros Manseng; AL: Alvarinho; VL: Verdelho; SA: Sauvignon; 8458: INRA-8458; GW: Gewurtzraminer; SH: Scheurebe; VE: Vermentino; RDU 2: INRA-ResDur 2; VO: Voltis; LL: Len de l’El; VJ: Verdejo; MU: Muscadelle. TPI: Total Polyphenol Index; α-NH_2_: Alpha-amine Nitrogen; Su: Sugar; NH_4_^+^: Mineral Nitrogen; TA: Titratable acidity. (m) = Must.

The prediction of the risk of protein instability is of particular interest to better anticipate the treatments to be carried out on musts or wines, and thus reduce their inconvenience [50]. To achieve this objective, a regression and classification tree (Figure 3) based on 268 observations with a mean ΔNTU of 50.7, was constructed. The variable “grape variety” allowed us to divide this trunk into six branches. These branches consist of a first group composed of Colombard and Mauzac (6.9 NTU), a second of Rkatsiteli, Chardonnay, Viognier, Riesling, Floréal, Arvine, and Ugni blanc (27.9 NTU), a third of Gros Manseng, Alvarinho, Verdelho (51.3 NTU), a fourth of Sauvignon, INRA-8458, Gewurtzraminer, Scheurebe, Vermentino, INRA-ResDur 2, Voltis (68.4 NTU), a fifth of Len de l’El (114.3 NTU), and finally a group composed of Verdejo and Muscadelle characterized by the highest level of protein instability (166.6 NTU). Apart from the group made up of Rkatsiteli, Chardonnay, Viognier, Riesling, Floréal, Arvine, and Ugni blanc, and the group composed of Verdejo and Muscadelle, whose limited number of observations did not allow for a further breakdown, the other groups have several ramifications. It should be noted that these branches are made up of quantitative variables essentially measured on the musts. The characterization of the must composition for each grape variety seems to be a good tool for predicting the risk of protein instability in wines.

The group made up of Sauvignon, INRA-8458, Gewurtzraminer, Scheurebe, Vermentino, INRA-ResDur 2, Voltis and that of Colombard and Mauzac contained wines whose protein instability was higher when must pH was also higher. For the last group, which had the largest population but the lowest protein instability, two additional sublevels could be observed. The first showed that musts with sugar levels between 17.1 and 18.5 °Brix gave the most unstable wines. The second indicates that musts with the lowest titratable acidity or NH_4_^+^ content produced the most unstable wines. For the Len de l’El, the most unstable wines were those from musts with the lowest alpha amino acid levels (<70.1 mg/L). Finally, the most unstable wines from the Gros Manseng, Alvarinho, and Verdelho group were those with the highest must total polyphenol index (>8.5).

These results show that it is possible to identify typologies of must likely to give more or less unstable wines with respect to protein haze. The grape variety is the first key qualitative factor to consider. Some grape varieties are known to give wines with a high instability and have been the subject of numerous studies such as Sauvignon [51], Chardonnay [12,52], Riesling [53], Manzoni Bianco [54], Macabeo [55], and Albarino [56]. Here, it should be noted that the white wines with the lowest protein instability came from an autochthonous variety of the southwest, Mauzac, and the most widely planted white grape variety in this wine region, Colombard. The latter has already demonstrated its good stability against haze formation [57]. These two varieties produce wines with a low risk of protein instability in the bottle, since under our conditions, their ΔNTU after heating was close to 2 NTU. At this level, a wine is generally considered stable [26] and does not require any bentonite addition to stabilize it. However, our study showed that the risk of instability increases as the pH of the must increases. This increase in must pH is likely to be a consequence of the climatic changes above-mentioned. This indicates that wines from these two grape varieties, which were not previously described as susceptible to cloudiness in the bottle, may become so in the future.

In contrast, Len de l’El, an indigenous grape variety from the southwest, has the highest protein instability behind Verdejo and Muscadelle, both of which are known to be unstable or at least to contain a high level of unstable protein [27,58,59]. The role of alpha amino acids raised earlier may reflect the climatic conditions during which the grapes ripened. Indeed, high temperatures and a limited vine water supply during berry development tend to reduce the amino acid content in the grapes [60].

Finally, wines from the Gros Manseng group, another indigenous grape variety, are more unstable when the total polyphenol index is high. The role of phenolic compounds in haze formation is known and it seems logical that such risk increases with the polyphenol content [13,61]. Several climatic factors such as temperature, exposure to UV radiation, and soil water availability during grape development can impact the level of polyphenol compounds found in musts and white wines (flavonols and proanthocyanidins) [62]. However, the relationship between climatic conditions during grape development and TPI values is not clear [44].

This study conducted on numerous grape varieties planted in the southwest region of France from four vintages tends to show that climate is an important factor in explaining the differences in protein instability measured in white wines. While it seems that hot and dry conditions during grape ripening are likely to favor instability, it is difficult to draw conclusions on whether the increased risk of instability is related to a change in the composition of the wine (notably an increase in pH) or to an increase in their unstable protein content. The interaction of the two factors cannot be excluded and deserve further investigation.

## Figures and Tables

**Table 1:** Information of musts and wines analyzed in the study (grape varieties, abbreviations, sample number, vintages, and regions).

Grape Variety	Abbreviation	Sample Number	Vintage
Gers			
Arvine	AR	2	2018
Scheurebe	SH	2	2018
Vermentino	VE	2	2018
Ugni blanc	UB	4	2018/2019
Chardonnay	CH	6	2017/2018/2019
Gewurztraminer	GE	6	2017/2019
Riesling	RI	6	2017/2018/2019
Floréal ^a^	FL	8	2018/2019
Voltis ^a^	VO	8	2018/2019
**Alvarinho**	AL	8	2016/2017/2018/2019
**INRA-8458**	8458	10	2016/2017/2018/2019
**Rkatsiteli**	RK	10	2016/2017/2018/2019
**Verdejo**	VJ	10	2016/2017/2018/2019
**Verdelho**	VL	10	2016/2017/2018/2019
INRA-ResDur 2 ^b^	RDU2	34	2018/2019
Sauvignon	SA	46	2016/2017/2018/2019
Gros Manseng	GM	88	2016/2017/2018/2019
Colombard	CO	148	2016/2017/2018/2019
Tarn			
Viognier	VI	4	2019
Muscadelle	MU	16	2017
Mauzac	MA	22	2016/2017
**Len de l’El**	LL	38	2016/2017/2018/2019
**Sauvignon**	SA	48	2016/2017/2018/2019

^a^ Resistant grape variety, INRA-ResDur 1; ^b^ Resistant grapevine variety, INRA-ResDur 2. In bold, grape varieties retained for the part: Impact of climate conditions on protein instability and basic must and wine parameters.

**Table 2:** Range and median values of oenological parameters.

	Must (*n* = 124)				Global Value	Wine (*n* = 124)				Global Value
	2016	2017	2018	2019	must (*n* = 268)	2016	2017	2018	2019	wine (*n* = 268)
Sugar (°Brix)	16.2 ^1^–23.8 ^2^; 17.6 ^3^ b	16.4–23.5; 19.7 a	15.9–24.6; 18.5 ab	16.7–24.3; 19.8 a	14.9–24.6; 19.7	-	-	-	-	-
TA (g/L tartaric acid)	4.26–10.29; 6.23 a	4.05–11.27; 6.72 a	4.89–9.92; 6.57 a	4.68–11.1; 7.21 a	3.33–11. 5; 6.8	4.28–8.45; 5.45 c	4.14–9.54; 5.94 ab	5.51–9.27; 7.1 a	5.01–9.09; 6.91 b	3.75–10.24; 6.67
pH	2.95–3.37; 3.16 ab	2.76–3.46; 3.07 c	2.82–3.33; 3.11 b	2.89–3.33; 3.2 a	2.6–3.66; 3.1	2.91–3.4; 3.11 a	2.83–3.76; 3.18 a	2.89–3.42; 3.05 a	2.81–3.6; 3.12 a	2.65–3.76; 3.1
TartA (g/L)	2.58–4.58; 3.57 a	1.93–6.38; 3.01 a	2.6–4.14; 3.2 a	2.03–4; 3.38 a	1.71–6.38; 3.26	1.1–3.35; 2.26 a	0.92–3.46; 1.81 a	1.22–2.73; 2.06 a	0.80–2.78; 1.93 a	0.80–4.39; 1.93
MA (g/L)	1.76–6.79; 4.04 c	2.58–8.09; 4.27 a	2.76–6.72; 4.34 bc	2.76–10.29; 5.62 ab	0.85–10.29; 4.31	1.45–4.56; 2.93 b	2.05–6.01; 3.02 a	2.11–6.29; 4.01 a	2.05–8.1; 4.16 a	0.43–8.10; 3.34
K^+^ (g/L)	1.09–1.59; 1.28 a	0.97–1.46; 1.18 a	0.97–1.55; 1.28 a	0.95–1.43; 1.22 a	0.43–1.88; 1.22	0.62–0.84; 0.7 a	0.48–0.77; 0.7 a	0.62–0.76; 0.7 a	0.61–0.78; 0.71 a	0.44–1.12; 0.7
TPI	5.7–10.5; 7.2 a	4.7–8; 6.2 b	4.8–8.4; 6.2 ab	4.4–8.3; 6 b	4.2–10.9; 6.6	4.5–9.2; 6.5 a	4.8–6.9; 5.4 b	4.4–8.4; 5.8 ab	4.3–7.6; 5.5 b	4.2–19.6; 5.9
α-NH_2_ (mg/L)	63.9–183.0; 133.1 b	74.5–234.2; 165.2 a	57.2–161.2; 131.1 b	83.3–232.1; 137.5 ab	41.2–298.5; 137.1	-	-	-	-	-
NH_4_^+^ (mg/L)	11.4–125.5; 61.8 b	41.3–102.4; 68.6 a	16–95; 56.1 b	31.7–164.5; 81.6 a	10.9–164.5; 62.3	-	-	-	-	-
Eth (% abv)	-	-	-	-		10.7–14.2; 11.4 b	11–14.4; 12 ab	10.5–15.3; 11.9 ab	9.7–14.9; 12.4 a	9.6–15.4; 12.1
VA (g/L acetic acid)	-	-	-	-		0.07–0.71; 0.14 a	0.11–0.63; 0.19 a	0.04–0.46; 0.17 a	0.09–0.46; 0.27 a	0.01–0.71; 0.2
SO_2_ F (mg/L)	-	-	-	-		15–28; 21 b	18–33; 24 b	16–42; 23 ab	17–40; 25 a	9.0–45.0; 23
SO_2_ T (mg/L)	-	-	-	-		56–132; 76 bc	69–121; 87 b	54–102; 74 c	82–134; 103 a	25.0–144.0; 89.4

^1^ Minimum; ^2^ Maximum; ^3^ Median; TA: Titratable acidity; TartA: Tartaric acid; MA: Malic acid; K^+^: Potassium; TPI: Total Polyphenol Index; α-NH_2_: Alpha-amine Nitrogen; NH_4_^+^: Mineral Nitrogen; Eth: Ethanol; VA: Volatile acidity; SO_2_ F: Free SO_2_; SO_2_ T: Total SO_2_. Different letters (a, b, c, ab, bc) indicates means significantly different by the Fisher test (*p* < 0.05).

**Table 3:** Pearson test correlation coefficients (*r*) among the oenological parameters of white must and wine (*p* < 0.05). Prot. Inst.: Proteins Instability; Brix: Sugar; TA: Titratable acidity; TartA: Tartaric acid; MA: Malic acid; α-NH_2_: Alpha-amine Nitrogen; NH_4_^+^: Mineral Nitrogen; K^+^: Potassium; TPI: Total Polyphenol Index; SO_2_ F: Free SO_2_; SO_2_ T: Total SO_2_; VA: Volatile acidity; Eth: Ethanol. In bold (Except *r*² × 100) are shown the coefficients for which a significant correlation is observed with a α = 0.05.

	Variables	Prot. Inst.	Brix	TA	pH	TartA	MA	α-NH_2_	NH_4_^+^	K^+^	TPI	TA	pH	SO_2_ F	SO_2_ T	TartA	MA	VA	K^+^	Eth	TPI	*r*²	*r*² × 100
Must	Brix	**0.160**	**1**																			0.026	2.6
	TA	**−0.536**	**−0.180**	**1**																		0.287	**28.7**
	pH	**0.544**	**0.148**	**−0.778**	**1**																	0.296	**29.6**
	TartA	0.022	−0.113	−0.115	−0.099	**1**																0.000	0.0
	MA	**−0.432**	**−0.125**	**0.882**	**−0.559**	**−0.452**	**1**															0.186	**18.6**
	α-NH_2_	−0.033	**0.252**	**0.130**	**0.288**	**−0.294**	**0.261**	**1**														0.001	0.1
	NH_4_^+^	**−0.136**	−0.072	**0.397**	−0.008	−0.078	**0.447**	**0.663**	**1**													0.018	1.8
	K^+^	**0.145**	−0.067	−0.055	**0.346**	**−0.227**	0.087	**0.254**	0.032	**1**												0.021	2.1
	TPI	**0.219**	**0.467**	**−0.127**	**0.159**	−0.073	−0.090	**0.189**	−0.101	**0.223**	**1**											0.048	4.8
Wine	TA	**−0.504**	−0.090	**0.892**	**−0.846**	−0.071	**0.767**	−0.052	**0.244**	**−0.200**	**−0.139**	1										0.254	**25.4**
	pH	**0.476**	**0.280**	**−0.579**	**0.796**	**−0.385**	**−0.274**	**0.440**	0.032	**0.369**	**0.278**	**−0.673**	**1**									0.227	**22.7**
	SO_2_ F	0.048	**0.175**	−0.034	0.115	0.005	0.012	**0.240**	**0.246**	−0.076	−0.067	−0.016	0.064	**1**								0.002	0.2
	SO_2_ T	0.065	**0.409**	0.006	**0.187**	**−0.304**	**0.157**	**0.413**	**0.308**	−0.094	0.086	−0.027	**0.255**	**0.576**	**1**							0.004	0.4
	TartA	**−0.179**	−0.107	**0.294**	**−0.468**	**0.656**	−0.047	**−0.193**	0.093	**−0.380**	0.000	**0.380**	**−0.607**	−0.021	**−0.231**	**1**						0.032	3.2
	MA	**−0.389**	**−0.171**	**0.817**	**−0.508**	**−0.457**	**0.944**	**0.235**	**0.394**	**0.196**	**−0.130**	**0.755**	**−0.249**	0.023	0.094	−0.096	**1**					0.152	**15.2**
	VA	−0.083	**0.653**	0.078	−0.037	**−0.198**	0.092	**0.314**	**0.138**	**−0.264**	**0.164**	0.112	**0.121**	**0.131**	**0.417**	−0.008	0.012	**1**				0.007	0.7
	K^+^	0.116	0.076	**−0.126**	**0.313**	**−0.274**	0.033	**0.337**	**0.176**	**0.247**	0.087	**−0.198**	**0.351**	0.071	**0.211**	**−0.377**	0.069	0.104	**1**			0.013	1.3
	Eth	**0.133**	**0.904**	**−0.199**	**0.170**	−0.082	**−0.156**	**0.208**	−0.091	0.017	**0.405**	**−0.126**	**0.279**	**0.208**	**0.338**	**−0.130**	**−0.197**	**0.599**	0.016	1		0.018	1.8
	TPI	−0.018	**0.395**	**0.134**	−0.057	**−0.259**	**0.192**	**0.219**	0.025	0.096	**0.742**	**0.145**	**0.149**	−0.099	**0.238**	−0.020	**0.164**	**0.253**	0.048	**0.267**	**1**	0.000	0.0

Numbers in bold represents the significant values.

## Data Availability

Not applicable.
